# Plasticity of plasma membrane compartmentalization during plant immune responses

**DOI:** 10.3389/fpls.2012.00181

**Published:** 2012-08-03

**Authors:** Susan L. Urbanus, Thomas Ott

**Affiliations:** Institute of Genetics, University of Munich,Martinsried, Germany

**Keywords:** heterogeneity, compartmentalization, membrane domains, membrane rafts, plant immunity, receptor–ligand interactions

## Abstract

Plasma membranes require high levels of plasticity to modulate the perception and transduction of extracellular and intracellular signals. Dynamic lateral assembly of protein complexes combined with an independent compositional lipid patterning in both membrane leaflets provide cells the opportunity to decorate this interface with specific proteins in an organized but dynamic manner. Such ability to dynamically reorganize the protein content of the plasma membrane is essential for the regulation of processes such as polarity of transport, development, and microbial infection. While the plant cell wall represents the first physical and mostly unspecific barrier for invading microbes, the plasma membrane is at the forefront of microbial recognition and initiation of defense responses. Accumulating evidence indicating dynamic compartmentalization of plasma membranes in response to environmental cues has increased the interest in the compositional heterogeneity of this bilayer. Here, we elucidate the recruitment of specific proteins into defined membrane structures that ensure functional compartmentalization of the bilayer during infection processes.

## INTRODUCTION

The interface between the cytoplasm and the outer environment in plant cells is comprised of the cell wall and the plasma membrane. In their fluid mosaic model, Singer and Nicolson (1972) proposed that biological membranes, such as the plasma membrane, consist of a phospholipid bilayered matrix that is randomly interspersed with integral proteins. Recent research on plasma membrane components has significantly refined this view. Accumulating evidence indicates that the plasma membrane consists of a phospholipid bilayer that contains dynamic membrane domains, some of which are enriched in sphingolipids, sterols and specific proteins and called membrane rafts (Brown and Rose, 1992; Simons and Ikonen, 1997; Xu et al., 2001). These membrane rafts are able to cluster into more stable (signaling) platforms upon the perception of certain stimuli and crosslinking (Kusumi et al., 2004; Hammond et al., 2005; Lingwood et al., 2008; Hogue et al., 2011). Most of the available knowledge and hypotheses on the function and formation of membrane rafts originates from studies in mammalian and yeast cells (Simson et al., 1995; Sheets et al., 1997; Simons and Ikonen, 1997; Malinska et al., 2003; Kusumi et al., 2004; Fan et al., 2010), where it has among others been shown that membrane rafts play an important role during microbial infection processes (Duncan et al., 2002; Lafont and van der Goot, 2005; van der Meer-Janssen et al., 2010). There is increasing evidence that membrane domains may be similarly involved in the interaction between microbes and plants (Zappel and Panstruga, 2008). Here we discuss the current view on plant plasma membrane remodeling in response to both symbiotic and pathogenic microbes.

## DYNAMIC MEMBRANE DOMAINS IN EUKARYOTIC CELLS

Since the *Keystone Symposium on Lipid Rafts and Cell Function*, the consensus definition of membrane rafts is that they are small (10–200 nm), heterogeneous, highly dynamic, sterol- and sphingolipid-enriched domains that compartmentalize cellular processes (Pike, 2006; Simons and Gerl, 2010). These rather elusive membrane rafts, due to their nanoscale size and dynamic behavior, can be stabilized through protein–protein, protein–lipid, and lipid–lipid interactions to form larger platforms that allow visualization by conventional light microscopy. Two main compartmentalizing forces seem to form membrane domains. The lipid raft model describes how sphingolipids can laterally associate with sterol molecules into close-packed assemblies (liquid-ordered phases; Simons and Ikonen, 1997; Lingwood and Simons, 2010). These close-packed, highly saturated rafts have different properties compared with the surrounding, less ordered and highly unsaturated phospholipid bilayer. Due to these different properties certain transmembrane and membrane-associated proteins preferentially insert into these rafts. In mammalian cells post-translational modifications such as myristoylation and palmitoylation were shown to target proteins preferentially to membrane rafts in the cytoplasmic membrane leaflet, while the addition of a glycosylphosphatidylinositol (GPI) moiety anchors proteins predominantly to membrane rafts at the outer leaflet (Schroeder et al., 1994; Varma and Mayor, 1998; Morrow et al., 2002; Zacharias et al., 2002). Evidence for lipid-dependency of membrane raft formation has been obtained from both artificial membrane models and living cells (Pralle et al., 2000; Hammond et al., 2005; Baumgart et al., 2007; Beck et al., 2007; Roche et al., 2008; Kaiser et al., 2009). It was also demonstrated that the cortical actin-based cytoskeleton plays a role in the compartmentalization of plasma membranes (Suzuki and Sheetz, 2001; Fujiwara et al., 2002; Kusumi et al., 2004; Lenne et al., 2006). Such impact, however, cannot be tested in artificial membrane models. In the membrane-skeleton fence and anchored-protein picket models the effect of the cytoskeleton on the lateral movement of membrane components is described (Kusumi and Sako, 1996; Sako et al., 1998; Fujiwara et al., 2002; Kusumi et al., 2004). The membrane-skeleton fence model proposes that the cytoplasmic domains of transmembrane and membrane-associated proteins can collide with the cytoskeletal filaments close to the membrane, thereby confining movement of these proteins to compartments that can vary in size from 30 to 250 nm. In the anchored-protein picket model, transmembrane proteins anchor to and line up along the cytoskeletal meshwork as pickets. Due to steric hindrance and hydrodynamic friction-like effects these proteins consequently limit diffusion of other membrane molecules in both membrane leaflets. Since both models affect membrane proteins as well as membrane lipids it can be assumed that dynamic compartmentalization of plasma membranes relies on an interplay between cytoskeleton-mediated and raft-derived effects. Indeed “hop-diffusion” where movement of proteins is temporarily confined by a cytoskeleton fence until the restrictive presence of the actin filaments is loosened by temporary breakdown of the filaments or an increase in the distance between the membrane and the cytoskeletal meshwork, has been described for a number of membrane raft proteins such as the human transferrin receptor (Sako and Kusumi, 1995; Kusumi et al., 2005). Membrane molecules can also move to neighboring compartments when they have gained sufficient kinetic energy to break through the restraining actin filament. The protein FORMIN-1 in *Arabidopsis thaliana* (thale cress) seems to be such a transmembrane picket protein, since it is able to anchor the actin cytoskeleton through the plasma membrane to the cell wall (Martiniere et al., 2011). However, experiments using the fluorescence recovery after photobleaching (FRAP) technique on fluorescently labeled membrane proteins in protoplasts that were re-growing their cell wall, demonstrate that in plants the constraining influence of the cell wall on the mobility of plasma membrane proteins is much greater than the influence of the cytoskeleton (Martiniere et al., 2012).

A frequently used, but much debated method in membrane raft research makes use of the differential packing of molecules in membranes to isolate close-packed assemblies from the plasma membrane through their greater resistance to detergents such as Triton (Heerklotz, 2002; Munro, 2003; Brown, 2006; Kierszniowska et al., 2009; Mongrand et al., 2010; Pathak and London, 2011; Tanner et al., 2011). Isolation of so called detergent-insoluble membranes (DIMs), or detergent-resistant membranes (DRMs), from *Nicotiana tabacum* (tobacco) demonstrated the enrichment of the sphingolipid glycosylceramide and the sterols stigmasterol, 24-methyl cholesterol, sitosterol, and cholesterol compared to detergent-soluble membrane (DSM) fractions. In contrast, relative amounts of phospholipids such as phosphatidylcholine and phosphatidylethanolamine are reduced in DIMs (Mongrand et al., 2004). Similar findings have been reported for DIMs prepared from *A. thaliana*, *Medicago truncatula* (barrel clover), *Phaseolus* vulgaris (common bean), and *Zea mays* (maize; Borner et al., 2005; Lefebvre et al., 2007; Furt et al., 2010; Carmona-Salazar et al., 2011). Proteome analyses of membrane fractions from the above mentioned plant species and additionally from *Solanum tuberosum* (potato), *Sinapis alba* (white mustard), and the monocotyledonous plant species *Avena sativa* (oat), *Secale cereale* (rye), and *Oryza sativa* (rice) demonstrate enrichment of proteins involved in different types of processes in DIMs, including signal transduction and abiotic and biotic stress responses (Shahollari et al., 2004; Morel et al., 2006; Fujiwara et al., 2009; Stanislas et al., 2009; Keinath et al., 2010; Krügel et al., 2012; Takahashi et al., 2012). There seems to be a preference for palmitoylation and myristoylation in proteins involved in signaling processes and stress responses enriched in DIMs, possibly targeting them to membrane rafts in the cytoplasmic membrane leaflet (Morel et al., 2006). Since DIM fractions may only partially, if at all, reflect the composition of individual membrane raft classes, localization of these putative raft-resident molecules in *in planta* membrane domains has to be validated by other techniques to allay concerns about artifactual membrane raft formation and co-purification of non-raft constituents. Additional imaging approaches of putative membrane rafts constituents and testing whether clustering of these putative raft-localized lipids, sterols or proteins is influenced by chemicals such as the sterol extracting methyl-β-cyclodextrin, support the proposed presence of such structures *in planta* (Krügel et al., 2008; Raffaele et al., 2009; Boutte et al., 2011). Furthermore, newly developed imaging techniques such as total internal reflection fluorescence microscopy (TIRFM) or variable-angle epifluorescence microscopy (VAEM) can be successfully applied to cell wall enclosed cells and allow the visualization of mobile proteins located in and around the plasma membrane with very high signal-to-background ratio (Owen et al., 2007; Konopka and Bednarek, 2008; Spira et al., 2012). A recent study showed TIRFM to also be applicable to study single molecule trafficking of PM-resident proteins in plant cells (Martiniere et al., 2012). These techniques will help to analyze domain structures in the plasma membrane in more detail in the future. Whether the plasma membrane domains formed in plant cells can always be categorized as membrane rafts remains to be fully elucidated.

## THE ROLE OF MEMBRANE DOMAINS DURING INFECTION PROCESSES

**FIGURE 1:**
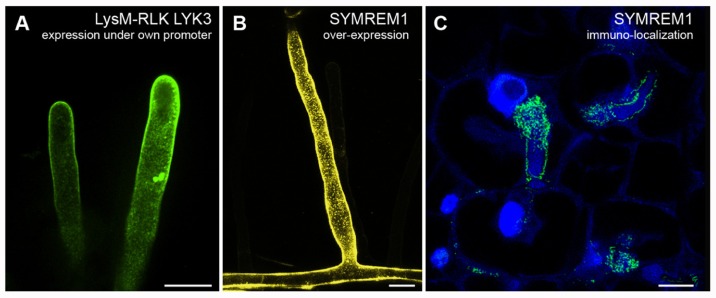
**Examples of membrane domain patterning in *Medicago truncatula***. **(A)** Root hair cells expressing GFP-tagged LysM-RLK MtLYK3 under the control of the native *LYK3* promoter in the kinase-inactive LYK3 mutant line *hcl-1*. Membrane domains as described earlier (Haney et al., 2011) can be observed with spinning-disk confocal microscopy. **(B)** Over-expression of the YFP-tagged remorin protein MtSYMREM1 in root hair cells results in the labeling of immobile domains in the plasma membrane that can be imaged with confocal laser scanning microscopy. **(C)** As originally reported (Lefebvre et al., 2010) similar domains, although smaller in size, can also be observed when immuno-localizing the MtSYMREM1 protein in the infection zone of mature root nodules using a specific MtSYMREM1 antibody. The MtSYMREM1 protein (green) resides on the infection thread membrane that surrounds invading rhizobial bacteria and accumulates at sites where these symbionts are released into the host cell. The plant and bacterial DNA is stained with DAPI (blue). Scale bars indicate 20 μm **(A,B)** and 1 μm **(C)**.

The integral membrane proteins FLOTILLIN-1 and FLOTILLIN-2 (synonymously called REGGIE-2 and REGGIE-1) are frequently used as markers for membrane rafts in the cytoplasmic leaflet of mammalian cells (Lang et al., 1998; Morrow et al., 2002; Blonder et al., 2004; Nixon et al., 2011). In plants and animals, members of this protein family were described to be involved in clathrin-independent endocytosis and are present on host-derived membranes surrounding intracellular microorganisms, indicating raft-mediated endocytosis as a possible entry point for microorganisms (Panter et al., 2000; Dermine et al., 2001; Saalbach et al., 2002; Glebov et al., 2006; Murphy et al., 2007; Li et al., 2008, 2012; Korhonen et al., 2012). Furthermore, evidence that flotillins are interacting and/or co-localizing with many signaling components, such as receptors and mitogen-activated protein kinases (MAPK), suggests that these proteins may act as scaffolds for a number of signaling processes (Langhorst et al., 2005; Haney et al., 2011; Amaddii et al., 2012). In *A. thaliana* a flotillin homolog was originally identified in DIMs and named AtFLOTILLIN-1 (AtFLOT1; Borner et al., 2005). Recently, a combination of confocal laser scanning microscopy, electron microscopy, and VAEM demonstrated that AtFLOT1 is present in distinct membrane domains, in clathrin-independent invaginations in the plasma membrane, and in endocytic vesicles with a size of approximately 100 nm (Li et al., 2012). Using transgenic plants expressing an artificial microRNA against AtFLOT1, the same study demonstrated that AtFLOT1 is required for meristem and seedling development. In *M. truncatula* MtFLOT2 and especially MtFLOT4 were found to be required for initiation of symbiotic infection structures (infection threads) and their elongation during interactions with the nitrogen-fixating bacterial symbiont *Sinorhizobium meliloti* (Haney and Long, 2010). Upon inoculation of *M. truncatula* roots with *S. meliloti* bacteria the evenly distributed fluorescently labeled MtFLOT4 punctae in the plasma membrane of a root hair cell start to accumulate at the root hair tip. These punctate structures might represent small clusters of membrane rafts that coalesce into a membrane raft platform at the root hair tip to facilitate the entry of the symbiont. Interestingly, fluorophore-tagged MtLYSIN-MOTIF-RECEPTOR-LIKE-KINASE-3 (MtLYK3), an RLK required for bacterial entry into the root hairs, localizes to mobile punctae in root hairs (**Figure 1A**) that become immobilized when the roots are inoculated with *S. meliloti* (Haney et al., 2011). Interestingly, fluorescently labeled MtFLOT4 and MtLYK3 punctae co-localize upon *S. meliloti* inoculation. In addition, the density of MtFLOT4 domains is decreased in the kinase-inactive MtLYK3 *hair curling-1* (*hcl-1*) mutant (Haney et al., 2011). These results suggest that these two proteins assemble into the same membrane domain upon perception of the symbiotic bacteria and possibly even interact with each other. Interestingly, the remorin protein *M. truncatula* SYMBIOTIC-REMORIN-1 (MtSYMREM1), a confirmed interactor of the LYK3 receptor, localizes to distinct domains when over-expressed in transgenic *M. truncatula* roots (**Figure 1B**). Such membrane patterns have also been found on nodular infection threads (**Figure 1C**), at bacterial release sites, and in symbiosome membranes as demonstrated by immuno-localizations and immunogold-labeling electron microscopy (Lefebvre et al., 2010).

Over the years, the plant-specific remorin proteins have become the most widely accepted raft marker proteins in plants. They are highly enriched in DIMs from several plant species (Mongrand et al., 2004; Shahollari et al., 2004; Morel et al., 2006; Lefebvre et al., 2007; Keinath et al., 2010; Krügel et al., 2012; Takahashi et al., 2012) and localize to methyl-β-cyclodextrin-sensitive domains of around 75 nm in the cytoplasmic membrane leaflet in *N. tabacum* cells, as demonstrated by statistical analysis of electron microscopy data (Raffaele et al., 2009). Over-expression of the potato remorin StREM1.3 leads to the labeling of large, immobile membrane domains that might resemble small clusters of membrane rafts (Raffaele et al., 2009) similar to those labeled by MtSYMREM1 (**Figure 1B**). While the biological function of remorins still remains unknown, members of this highly diverse multi-gene family have been shown to regulate viral spreading in leaves (Raffaele et al., 2009), interactions between *M. truncatula* and *S. meliloti* during root nodule symbiosis (Lefebvre et al., 2010), and to serve potential roles during plant–pathogen interactions (Jarsch and Ott, 2011). Additionally, their ability to oligomerize and to interact with signaling proteins such as the symbiotic receptors and the negative regulator of immune responses RPM1-INTERACTING-PROTEIN-4 (RIN4), suggests functions as scaffolding proteins during signal transduction (Liu et al., 2009; Lefebvre et al., 2010; Toth et al., 2012). In *A. thaliana* suspension cells the group 1 remorin AtREM1.3 is phosphorylated in the intrinsically disordered N-terminal domain within 10 min of incubation with flg22, a conserved 22 amino acid peptide from the bacterial-derived elicitor flagellin (Benschop et al., 2007; Marín and Ott, 2012), suggesting close functional dependency of AtREM1.3 on the functional receptor-like kinase AtFLAGELLIN-SENSITIVE-2 (FLS2). In analogy to MtLYK3, flg22-dependent reduction in the lateral mobility of fluorescently tagged AtFLS2 was observed in *A. thaliana* protoplasts when analyzed by FRAP (Ali et al., 2007). These data suggest that AtFLS2 becomes part of a larger, less mobile complex upon ligand-binding and/or becomes confined to membrane rafts upon ligand-binding. The concept of ligand-dependent recruitment to membrane rafts is supported by the fact that FLS2 is highly recruited to DIMs upon flg22 treatment of *A. thaliana* suspension cells, as demonstrated by quantitative mass spectrometric analyses (Keinath et al., 2010). These findings together with FLS2 being endocytosed upon flg22 treatment in young leaves (Robatzek et al., 2006), support possible links between localization of signaling proteins in membrane domains and endocytic events.

The functional importance of membrane domains during infection processes is also underlined by results from a number of studies on interactions of plants with pathogenic oomycetes or fungi. The NADPH oxidase RESPIRATORY-BURST-OXIDASE-HOMOLOG-D (RBOHD)-mediated production of reactive oxygen species is one of the first signaling responses initiated upon the perception of the oomycete-derived elicitor cryptogein (Simon-Plas et al., 2002). NtRBOHD-mediated H_2_O_2_-generation was detected in small patches along the plasma membrane in *N. tabacum* cells using transmission electron microscopy after staining with CeCl_3_ (Lherminier et al., 2009). A possible membrane raft localization of NtRBOHD is also supported by its identification in DIMs from *N. tabacum* after treatment with cryptogein, together with its negative regulator RAC/ROP GTPase NtRAC5 (Mongrand et al., 2004; Morel et al., 2006). *In planta* evidence for dynamic compartmentalization of membrane proteins was also reported upon host cell infection of *Hordeum vulgare* (barley) and *A. thaliana* by the powdery mildew fungus *Blumeria graminis*. Focal accumulations around fungal entry sites were observed for three otherwise evenly distributed fluorescently labeled proteins all involved in powdery mildew penetration resistance, namely MILDEW-RESISTANCE-LOCUS-O (MLO), the syntaxin HvREQUIRED-FOR-MLO-SPECIFIED-RESISTANCE-2 (HvROR2) and its ortholog AtPENETRATION-1 (AtPEN1; Bhat et al., 2005). These focal accumulations may represent membrane raft platforms, especially since they coincide with higher levels of sterols as demonstrated by filipin staining. The host-derived extrahaustorial membrane encasing the haustorial feeding structure of successfully entered fungi or oomycetes is clearly distinct from invaginated extensions of the plasma membrane, as demonstrated by the exclusion of several plasma membrane markers (Koh et al., 2005; Micali et al., 2011; Lu et al., 2012). However, differential presence of plasma membrane proteins such as AtFLS2, AtPEN1, and StREM1.3 in extrahaustorial membranes implies a mechanism to be in place that can actively determine whether proteins are included into the extrahaustorial membrane (Lu et al., 2012). Membrane reorganization may also be triggered and/or enhanced by changes in the lipid patterning of plasma membranes, as the polyphosphoinositide phosphatidylinositol-3-phosphate (PI3P) can be directly targeted by certain fungal and oomycete effectors during plant cell entry (Kale et al., 2010). Polyphosphoinositides have been shown to be enriched in *N. tabacum* DIMs and electron microscopy of immunogold-labeled phosphatidylinositol-4,5-bisphosphate (PI4,5P_2_) revealed the presence of distinct domains of approximately 25 nm in the plasma membrane (Furt et al., 2010). However, these domains are not methyl-β-cyclodextrin-sensitive, indicating that these domains are not membrane rafts according to the consensus definition.

## CONCLUSIONS AND PERSPECTIVE

Although the concept of membrane rafts still remains to be unequivocally proven in plants, several lines of research clearly demonstrate that the plant plasma membrane is dynamically compartmentalized during biological processes such as infection of host cells by microorganisms. Differently sized domains that could resemble single membrane rafts, small clusters of membrane rafts, large membrane raft platforms, or other types of membrane domains beyond the membrane raft concept, can be observed in the plasma membrane with electron and/or conventional light microscopy. Current data suggest that there is not one but multiple types of membrane domains, that host multimeric signaling complexes which specifically assemble within the plasma membrane in a stimulus-dependent manner. Minor changes in lipid or protein aggregation, such as ligand–receptor complex formation, could induce the assembly of an appropriate membrane domain that can transduce the changing environmental conditions. The presence of signaling components in membrane rafts or other distinct domains may potentially modulate their activity and enhance the interactions between domain-resident components, while reducing interactions with non-domain components. A clear correlation between endocytosis and signaling is apparent in many of the discussed examples, arguing for the concept of membrane rafts serving as signaling hubs that can be exploited by (facultative) intracellular microbes to successfully establish themselves in their host. To gain further knowledge on the behavior of these dynamic plasma membrane domains *in planta*, imaging techniques with high spatial and/or temporal resolution are needed that go beyond conventional light microscopy. For example, electron microscopy and super-resolution fluorescent microscopy could be used to determine the size of plasma membrane domains in fixed samples, Förster resonance energy transfer (FRET)-based live-cell imaging to determine interactions between residents of these domains, and TIRFM or VAEM to visualize the dynamics of these domains in living cells with intact cell wall.

## Conflict of Interest Statement

The authors declare that the research was conducted in the absence of any commercial or financial relationships that could be construed as a potential conflict of interest.
